# Timing of specialist palliative care and acute healthcare utilization at the end of life among adults who died of cancer: a nationwide cohort study

**DOI:** 10.1186/s12885-026-16077-0

**Published:** 2026-04-28

**Authors:** Nelli-Sofia Nåhls, Hanna-Riikka Lehto, Satu Ahtiluoto, Mikko Nuutinen, Harriet Finne-Soveri, Tiina Saarto, Timo Carpén

**Affiliations:** 1 Department of Oncology, Vaasa Central Hospital, The Wellbeing services County of Ostrobothnia, Hietalahdenkatu 2-4, Vaasa, 60130 Finland; 2 Department of Oncology, Comprehensive Cancer Center, Helsinki University Hospital, Helsinki, Finland; 3 Department of General Medicine, Division of Palliative Medicine, University of Turku, Turku, Finland; 4 Palliative Care Center, Comprehensive Cancer Center, Faculty of Medicine, Helsinki University Hospital, University of Helsinki, Helsinki, Finland; 5 Nordic Healthcare Group, Helsinki, Finland; 6 The Department of Healthcare and Social Welfare, Finnish Institute for Health and Welfare, Helsinki, Finland

**Keywords:** Cancer, Specialist palliative care, End-of-life care, Healthcare utilization, Hospitalization

## Abstract

**Background:**

Cancer is one of the leading causes of death worldwide, with high healthcare utilization in the last months of life. Specialist palliative care (SPC) may reduce unnecessary healthcare utilization, but nationwide evidence on timing is limited. This nationwide cohort study examined whether the timing of SPC was associated with end-of-life healthcare utilization among adults who died of cancer in Finland.

**Methods:**

We conducted a nationwide retrospective cohort study using register data. All adults who died of cancer in 2019 were identified from the national Causes of Death Register (*n* = 12879). Timing of first SPC contact was categorized as first SPC contact > 30 days before death, first SPC contact ≤ 30 days before death, or no SPC contact. Outcomes included emergency department (ED) contacts, hospitalizations, hospital readmissions, use of SPC services in the last 30 days of life, and place of death. Analyses were stratified analyses by age, sex, and municipality type.

**Results:**

Overall, 2617 patients (20%) had first SPC contact > 30 days before death, 1256 (10%) had first SPC contact ≤ 30 days before death, and 9006 (70%) had no SPC contact. Compared with patients with no SPC contact, those with first SPC contact > 30 days before death had fewer ED contacts (46% vs. 57%), fewer secondary care hospitalizations (26% vs. 53%), and fewer secondary care readmissions (8% vs. 20%). In stratified analyses by age, sex, and municipality type, first SPC contact > 30 days before death was consistently associated with lower acute healthcare utilization. Patients with first SPC contact > 30 days before death and those with first SPC contact ≤ 30 days before death were less likely to die in hospital than those with no SPC contact.

**Conclusions:**

Initiation of SPC more than 30 days before death was associated with reduced acute healthcare utilization and fewer hospital deaths, supporting integration of SPC into cancer care.

## Background

Cancer is a major cause of mortality in Western countries, accounting for more than one in five deaths [1]. The final months of life for patients with cancer are often characterized by intensive healthcare use, including frequent emergency department (ED) visits, hospitalizations, and readmissions. Such patterns are considered indicators of aggressive end-of-life (EOL) care and have been associated with increased costs and potentially poorer quality of care [2–5].

Specialist palliative care (SPC) aims to relieve complex symptoms, support patients and families, and coordinate care across settings [6]. Randomized and observational studies have consistently shown that early integration of SPC improves quality of life (QoL), reduces symptom burden, and decreases aggressive medical interventions near death [7–12]. In addition, early SPC initiation has been linked with reduced acute hospital utilization and lower health care costs [13–19]. Despite this evidence, SPC remains underused, and referrals are often late, limiting its potential benefit [8, 20–24].

In addition to underuse, access to SPC is influenced by limited resources and the lack of universally consistent referral criteria. To ensure sustainability, palliative care should be delivered primarily by generalist healthcare providers, with SPC focused on patients with more complex needs [25, 26].

Population-level data on the impact of palliative care timing is still limited. In the Netherlands, Boddaert et al. reported that only 16% of cancer patients initiated SPC more than 30 days before death, highlighting the persistently late timing of referrals [22]. In Finland, Ahtiluoto et al. investigated SPC use across both malignant and non-malignant conditions and found that only around 30% of individuals who died from cancer received SPC [27]. Smaller hospital-based studies further suggest that longer SPC involvement may reduce hospitalizations and support more appropriate EOL care [19, 28–30]. However, comprehensive nationwide evidence on EOL healthcare use specifically among individuals with cancer as the underlying cause of death is still lacking, and no prior studies have evaluated the impact of SPC timing on such a population.

Therefore, we conducted a nationwide, register-based cohort study including all adults who died of cancer to examine whether initiation of SPC more than 30 days before death is associated with acute healthcare utilization (ED contacts, hospitalizations, and readmissions), use of SPC services, and place of death.

## Materials and methods

### Cohort selection

The study cohort included all 12,879 adult patients who died of cancer in Finland in 2019. Patients were identified nationwide from the 2019 Causes of Death Register (Statistics Finland) using International Classification of Diseases, 10th Revision (ICD-10) codes indicating malignant neoplasms (C00–C97). Initially, 12,890 patients fulfilled the inclusion criteria; however, 11 patients who died outside Finland were excluded, resulting in a final study population of 12,879 patients. The study was reported in accordance with the Strengthening the Reporting of Observational Studies in Epidemiology (STROBE) guidelines [31].

### Data collection

Data was retrieved from two comprehensive nationwide administrative databases: the National Care Register, which compiles data on healthcare contacts from all public and private healthcare providers, and Kanta Services, a legally mandated digital platform that integrates information from social welfare and healthcare sectors. By linking these registries via health service unit codes, detailed data were obtained regarding patient demographics, healthcare utilization, social care service utilization, and use of SPC. Data collection covered the period from the beginning of 2018 until the end of 2019. Further information on data collection processes has been published previously [27]. Registry data for each patient was linked using each patient’s personal identification code. After the linkage, the data was pseudonymized.

### Utilization of health and social care services

Finnish healthcare is publicly funded and comprises primary, secondary, and tertiary care services. Primary healthcare services are provided by municipalities, while specialized medical care is offered by secondary and tertiary care hospitals. Secondary care is provided by 20 hospital district hospitals, and tertiary care by five university hospitals. For analytical purposes, secondary and tertiary care services were combined and referred to collectively as “secondary care.” In Finland, cancer care is provided exclusively within secondary care.

### Specialist palliative care contact

Palliative care services in Finland are provided at both general and specialist levels. This study focused specifically on SPC, defined as care provided by palliative care specialists across various settings, including specialized outpatient clinics, hospital-at-home services, specialized inpatient units or hospices, and inpatient consultations by palliative care specialists. In this study, SPC inpatient units included hospices. ICD-10 code Z51.5 indicates a documented palliative care decision, marking the transition from disease-modifying treatment to palliative intent; the first occurrence in the Care Register was considered the date of that decision.

Patients were categorized according to the timing of their first SPC contact (> 30 days before death vs. ≤30 days before death vs. no SPC contact). The 30-day threshold was chosen based on prior evidence indicating that more than 30 days of SPC involvement is generally required to achieve meaningful benefits [2, 3, 22, 23]. This cutoff is also aligned with widely used EOL quality indicators developed by Earle and colleagues, who identified healthcare use in the last 30 days of life, including ED visits, hospitalizations, and intensive care unit admissions, as markers of overly aggressive care [2, 3, 32].

### Place of death

Information on the place of death was derived from the 2019 Causes of Death Register. Place of death was classified into four categories: home, hospital (including primary healthcare hospitals and secondary care hospitals), long-term care facilities, and specialized palliative care inpatient unit.

### Ethical statement

The study was conducted at the Finnish Institute for Health and Welfare (THL) as part of the Project on Quality Information on Palliative Care and End-of-life Care. The study protocol was approved by the Finnish Institute for Health and Welfare (THL; decision number 12345556). This was a nationwide retrospective register-based study including only deceased individuals and involving no human interventions. In accordance with the Act on the Secondary Use of Social and Health Information (522/2019), separate ethics committee approval and informed consent were not required. All methods were carried out in accordance with relevant guidelines and regulations.

### Statistical analyses

All statistical analyses were conducted using IBM SPSS Statistics version 29 (IBM Corp, Armonk, NY, USA). Descriptive statistics are presented as medians (ranges) or as frequencies and percentages. Patients were categorized according to the timing of their first SPC contact (> 30 days before death vs. ≤30 days before death vs. no SPC contact). Pearson’s chi-squared test and Fisher’s exact test were used to compare categorical variables between groups. Mann-Whitney U tests were employed to analyze continuous variables, such as hospital days, due to non-normal distributions. Descriptive analyses were performed to examine the distribution of ED contacts and hospitalizations in secondary care during the last month of life according to timing of SPC initiation. The analyses were stratified by age (< 75 vs. ≥75 years), gender, and municipality type (urban, semi-urban, rural). Odds ratios with 95% confidence intervals were calculated, using “no SPC contact” as the reference category. A *p*-value < 0.05 was considered statistically significant. Municipality type (urban, semi-urban, rural) was defined according to the classification of Statistics Finland, based on the proportion of the population living in urban settlements and population density [33].

## Results

### Patient characteristics

**Fig. 1:**
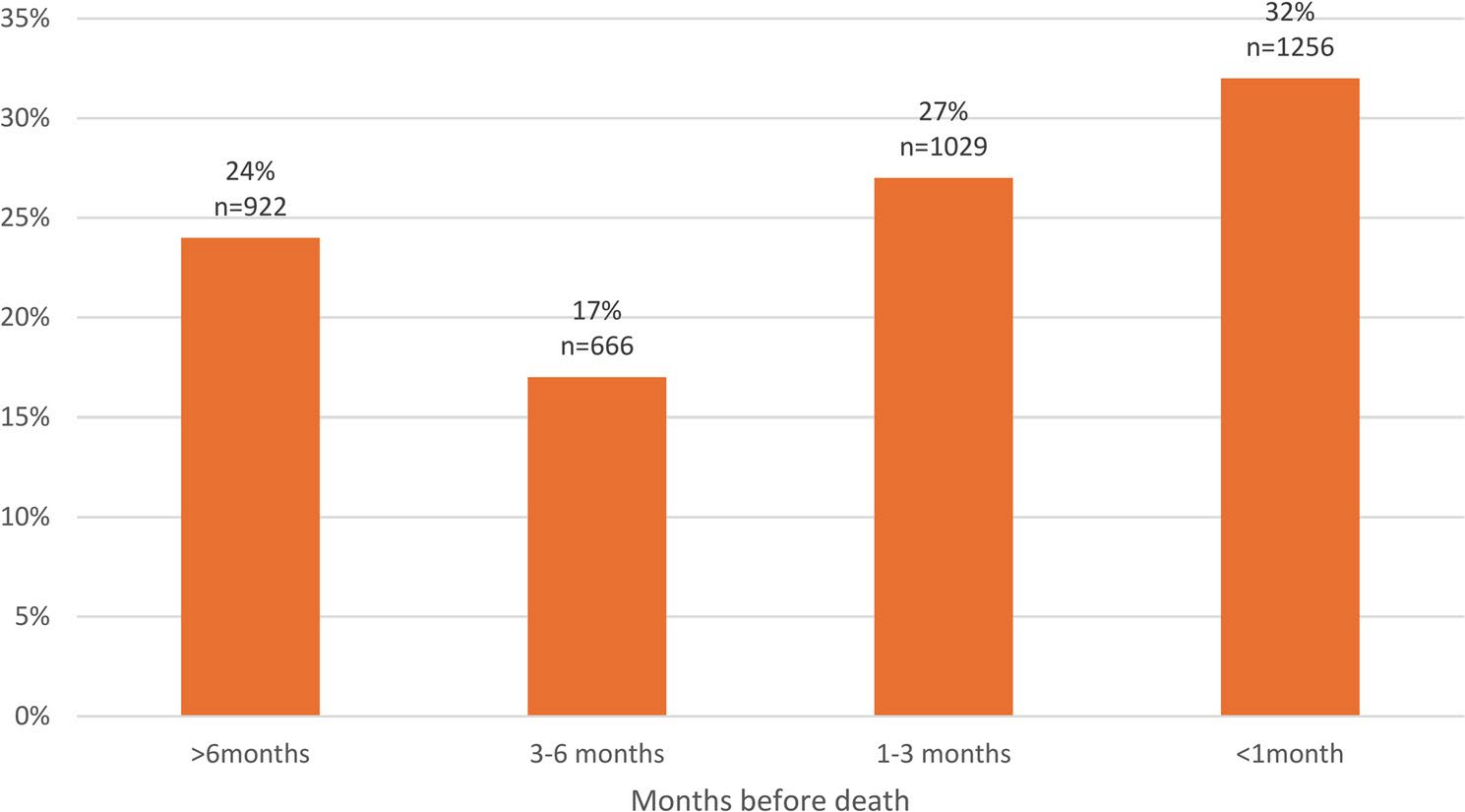
Timing of the first specialist palliative care contact before death among patients with cancer who received SPC

The study cohort consisted of 12879 patients who died of cancer in Finland in 2019. Median age at death was 75 years (range 18–105), and 54% of patients were male. Overall, 3873 patients (30%) had at least one SPC contact; 2617 patients (20%) had first SPC contact > 30 days before death, 1256 patients (10%) had first SPC contact ≤ 30 days before death, and 9006 patients (70%) had no SPC contact. The median interval from first SPC contact to death was 122 days among patients with first SPC contact > 30 days before death and 12 days among patients with first SPC contact ≤ 30 days before death. Compared with patients with no SPC contact, those with first SPC contact > 30 days before death were more often residents of urban municipalities (82% vs. 58%, *p* < 0.001) and more frequently had documentation of ICD-10 code Z51.5, indicating a palliative care decision (80% vs. 43%, *p* < 0.001) (Table 1; Fig. 1). 

**Table 1:** Patient characteristics

Variable	All patients (*n* = 12879)	First SPC contact > 30 days before death (*n* = 2617)	First SPC contact ≤ 30 days before death (*n* = 1256)	No SPC contact (*n* = 9006)
Age in years median (range)	75 (18–105)	75 (18–104)	73 (21–100)	76 (18–105)
Gender, n (%)				
Male	6913 (54)	1367 (52)	666 (53)	4880 (54)
Female	5966 (46)	1250 (48)	590 (47)	4126 (46)
Municipality type, n (%)*				
Urban	8403 (65)	2144 (82)	1048 (83)	5211 (58)
Semi-urban	2295 (18)	232 (9)	98 (8)	1965 (22)
Rural	2175 (17)	240 (9)	109 (9)	1826 (20)
ICD-10 Diagnosis code Z51.5 Palliative care, n (%)	6933 (54)	2095 (80)	933 (74)	3905 (43)
Specialist palliative care contact, n (%)	3873 (30)	2617 (100)	1256 (100)	0 (0)
Median time from the first SPC contact to the death (IQR)	64 days (IQR 151)	122 days (IQR 182)	12 days (IQR 15)	-
Cancer diagnoses, n (%)				
Upper gastrointestinal cancer	2756 (21)	606 (23)	326 (26)	1824 (20)
Lung cancer	2342 (18)	432 (17)	220 (18)	1690 (19)
Breast cancer	881 (7)	205 (8)	83 (7)	593 (7)
Colorectal cancer	1445 (11)	339 (13)	131 (10)	975 (11)
Prostate cancer	921 (7)	215 (8)	61 (5)	645 (7)
Gynecologic cancer	735 (6)	160 (6)	81 (6)	494 (6)
Cancer of the urinary tract	706 (6)	132 (5)	64 (5)	510 (6)
Lymphomas	560 (4)	68 (3)	48 (4)	444 (5)
Invasive skin cancers	277 (2)	40 (2)	39 (3)	198 (2)
Primary CNS malignancies	373 (3)	81 (3)	21 (2)	271 (3)
Head and neck cancer	281 (2)	67 (3)	26 (2)	188 (2)
Sarcomas	119 (1)	34 (1)	15 (1)	70 (1)
Leukemia	355 (3)	49 (2)	28 (2)	278 (3)
Myeloma	278 (2)	40 (2)	25 (2)	213 (2)
Other	850 (7)	149 (6)	88 (7)	613 (7)

*Abbreviations*: *n*  number of patients, *SPC*  Specialist palliative care, *CNS* Central nervous system

*For 6 (0.1%) patients, municipality was a foreign country

### Healthcare utilization during the last six months of life

In the final six months of life, healthcare resource utilization was substantial across all patients: 89% had ED contacts, 78% were hospitalized in secondary care, and 63% in primary care. More than half (58%) of patients received home care, and 14% used social care services. 

### Healthcare utilization in the last month of life according to SPC timing

**Fig. 2:**
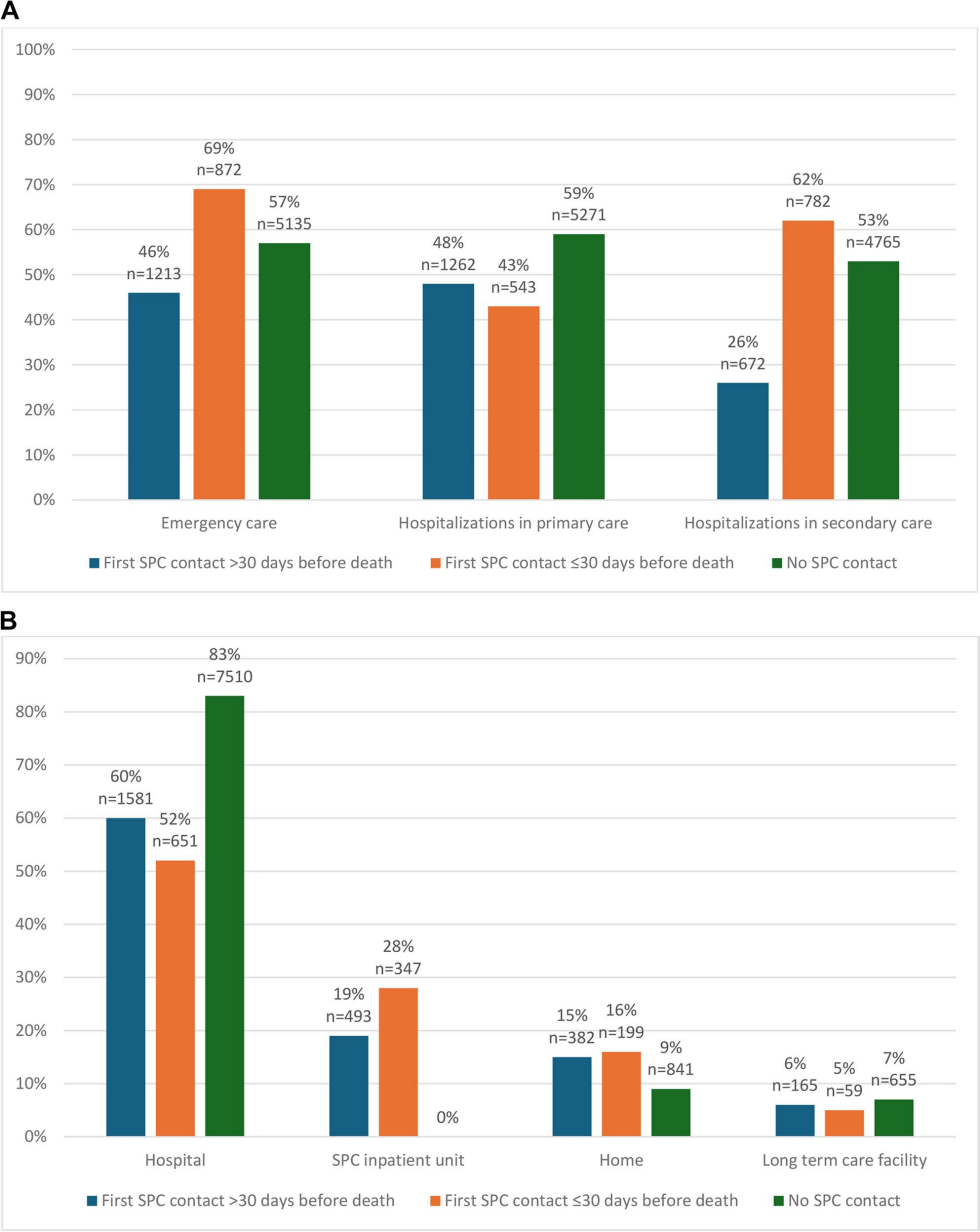
**A** Emergency department contacts and hospitalizations in the last month of life by timing of first specialist palliative care contact (SPC): first SPC contact >30 days before death, first SPC contact ≤30 days before death, and no SPC contact. **B** Place of death by timing of first specialist palliative care (SPC) contact: first SPC contact >30 days before death, first SPC contact ≤30 days before death, and no SPC contact

Compared with patients with no SPC contact, those with first SPC contact >30 days before death had significantly fewer ED contacts during the last month of life (46% vs. 57%, *p*<0.001). In contrast, patients with first SPC contact ≤30 days before death had more ED contacts than those with no SPC contact (69% vs. 57%, *p*<0.001) (Table 2, Figure 2A).

**Table 2:** Healthcare utilization in the last month of life by SPC contact

Variable	All (%) (*n* = 12879)	First SPC contact > 30 days before death (*n* = 2617)^a^	First SPC contact ≤ 30 days before death (*n* = 1256)^b^	No SPC contact (9006)^c^	*P*-value
Emergency department contacts, n (%)	7221 (56)	1214 (46)	872 (69)	5135 (57)	<0.001^ab^/<0.001^bc^/<0.001^ac^
Outpatient clinic contacts, n (%)					
Secondary care	6741 (52)	894 (34)	853 (68)	4994 (56)	<0.001^ab^/<0.001^bc^/<0.001^ac^
Primary health care	8561 (67)	1622 (62)	888 (71)	6051 (67)	< 0.001^ab^/0.013^bc^/<0.001^ac^
Cancer clinic	3459 (27)	665 (25)	505 (40)	2534 (28)	<0.001^ab^/<0.001^bc^/<0.001^ac^
Hospitalizations, n (%)					
Secondary care	6219 (48)	672 (26)	782 (62)	4765 (53)	<0.001^ab^/<0.001^bc^/<0.001^ac^
Primary health care	7076 (55)	1262 (48)	543 (43)	5271 (59)	0.004^ab^/<0.001^bc^/<0.001^ac^
Cancer inpatient unit	1544 (12)	179 (7)	234 (19)	1131 (13)	<0.001^ab^/<0.001^bc^/<0.001^ac^
Readmissions, n (%)					
Emergency department recontacts	3264 (25)	555 (21)	444 (35)	2265 (25)	<0.001^ab^/<0.001^bc^/<0.001^ac^
Secondary care hospital readmission	2310 (18)	201 (8)	295 (24)	1814 (20)	< 0.001^ab^/0.006^bc^/<0.001^ac^
Primary care hospital readmissions	1691 (13)	270 (10)	127 (10)	1294 (14)	0.843^ab^/<0.001^bc^/<0.001^ac^
Specialist palliative care, n (%)					
Palliative care outpatient clinic	1101 (9)	565 (22)	536 (43)	0 (0)	<0.001^ab^/<0.001^bc^/<0.001^ac^
Hospital-at-home	1627 (13)	1072 (41)	555 (44)	0 (0)	0.057^ab^/<0.001^bc^/<0.001^ac^
Special palliative care inpatient unit	921 (7)	548 (21)	373 (30)	0 (0)	<0.001^ab^/<0.001^bc^/<0.001^ac^
Social services, n (%)	1439 (11)	276 (11)	98 (8)	1065 (12)	0.007^ab^/<0.001^bc^/0.071^ac^
Home care, n (%)	5246 (41)	1306 (50)	579 (46)	3361 (37)	0.027^ab^/<0.001^bc^/<0.001^ac^
Place of death, n (%)					
Hospital	9742 (76)	1581 (60)	651 (52)	7510 (83)	<0.001^ab^/<0.001^bc^/<0.001^ac^
Specialist palliative care inpatient unit	840 (7)	493 (19)	347 (28)	-	<0.001^ab^/<0.001^bc^/<0.001^ac^
Home	1422 (11)	382 (15)	199 (16)	841 (9)	0.309^ab^/<0.001^bc^/<0.001^ac^
Long term care facility	879 (7)	165 (6)	59 (5)	655 (7)	0.045^ab^/<0.001^bc^/0.089^ac^

Superscripts indicate pairwise group comparisons (a vs. b, b vs. c, a vs. c), with corresponding p-values reported in the table

*Abbreviations*: *n*  number of patients, *SPC* Specialist palliative care

Hospitalizations showed similar patterns. Patients with first SPC contact >30 days before death had fewer secondary care hospitalizations (26% vs. 53%, *p*<0.001), whereas patients with first SPC contact ≤30 days before death had a higher rate (62% vs. 53%, *p*<0.001) compared with patients with no SPC contact. Similarly, primary care hospitalizations were less common among patients with first SPC contact >30 days before death than among those with no SPC contact (48% vs. 59%, *p*<0.001).

Cancer clinic outpatient visits were less frequent among patients with first SPC contact >30 days before death than among those with no SPC contact (25% vs. 28%, *p*<0.001), whereas a higher proportion of patients with first SPC contact ≤30 days before death had cancer clinic contacts (40% vs. 28%, *p*<0.001). Cancer inpatient unit hospitalizations were also less common among patients with first SPC contact >30 days before death (7% vs. 13%, *p*<0.001) and more common among patients with first SPC contact ≤30 days before death (19% vs. 13%, *p*<0.001) compared with patients with no SPC contact.

### Hospital readmissions

Patients with first SPC contact >30 days before death had significantly fewer readmissions than those with no SPC contact: ED recontacts (21% vs. 25%, *p*<0.001) and secondary care readmissions (8% vs. 20%, *p*<0.001). Patients with first SPC contact ≤30 days before death had more ED recontacts (35% vs. 25%, *p*<0.001) and more secondary care readmissions (24% vs. 20%, p=0.006) than those with no SPC contact (Table 2).

### Stratified analyses

Stratified analyses showed that first SPC contact ≤30 days before death was associated with higher odds of ED contacts across all subgroups, including age, gender, and municipality type (ORs 1.433-2.024). In contrast, first SPC contact >30 days before death was consistently associated with reduced ED contacts (ORs 0.559-0.771). Patterns were similar for secondary care hospitalizations. First SPC contact ≤30 days before death was associated with increased hospitalization risk (ORs 1.246-2.502), whereas first SPC contact >30 days before death was associated with substantially lower odds (ORs 0.280-0.353) across all subgroups (Table 3).

**Table 3:** Distribution of emergency department contact and hospitalization in secondary care during the last month of life by timing of first SPC contact (>30 days before death, ≤30 days before death, no SPC contact), stratified by age (<75 vs ≥75 years), gender, and municipality type

A. Emergency department contacts							
SPC category	Age <75 years OR (95% CI) (*n*=6075)	Age ≥75 years OR (95% CI) (*n*=6802)	Male OR (95% CI) (*n*=6913)	Female OR (95% CI) (*n*=5966)	Urban OR (95% CI) (*n*=8403)	Semi-urban OR (95% CI) (*n*=2295)	Rural OR (95% CI) (*n*=2175)
No SPC contact (ref)	1.00	1.00	1.00	1.00	1.00	1.00	1.00
First SPC contact ≤30 days before death	1.433 (1.205-1.704) ***	2.014 (1.669-2.431) ***	1.482 (1.246-1.763) ***	2.024 (1.679-2.439) ***	1.602 (1.389-1.848) ***	2.159 (1.374-3.393) ***	1.556 (1.041-2.327) **
First SPC contact >30 days before death	0.655 (0.577-0.744) ***	0.641 (0.568-0.724) ***	0.628 (0.557-0.709) ***	0.685 (0.603-0.778) ***	0.615 (0.556-0.681) ***	0.559 (0.424-0.738) ***	0.771 (0.589-1.010)

B. Secondary care hospitalizations							
No SPC contact (ref)	1.00	1.00	1.00	1.00	1.00	1.00	1.00
First SPC contact ≤30 days before death	1.324 (1.115-1.572) ***	1.502 (1.262-1.788) ***	1.502 (1.268-1.778) ***	1.445 (1.213-1.721) ***	1.246 (1.087-1.427) **	2.502 (1.614-3.880) **	1.539 (1.040-2.278) *
First SPC contact >30 days before death	0.280 (0.245-0.321) ***	0.318 (0.276-0.366) ***	0.284 (0.248-0.324) ***	0.339 (0.294-0.390) ***	0.271 (0.243-0.303) ***	0.319 (0.231-0.440) ***	0.353 (0.259-0.480) ***

*Abbreviations*: *SPC* Specialist palliative care, *OR* Odds ratio, *CI* Confidence interval

*p<0.05, **p<0.01, ***p<0.001

### Use of SPC services

Use of SPC services differed between patients with first SPC contact >30 days before death and those with first SPC contact ≤30 days before death. Patients with first SPC contact ≤30 days before death had more frequent use of outpatient SPC clinics (43% vs. 22%) and SPC inpatient units (30% vs. 21%), whereas no significant difference was observed in the use of hospital-at-home services (44% vs. 41%).

### Place of death

Place of death varied substantially between the SPC categories. Patients with first SPC contact >30 days before death were less likely to die in hospital (60%) than those with no SPC contact (83%, *p*<0.001). Among patients who received SPC, both patients with first SPC contact >30 days before death and those with first SPC contact ≤30 days before death had notable proportions of deaths occurring in SPC inpatient units (19% and 28%, respectively). Deaths in long-term care facilities were similar between the two SPC timing categories (6% among patients with first SPC contact >30 days before death and 5% among those with first SPC contact ≤30 days before death) (Table 2, Figure 2B).

## Discussion

In this nationwide cohort of adult patients who died of cancer in Finland in 2019, first SPC contact > 30 days before death was associated with reduced acute healthcare utilization during the last month of life. Patients with first SPC contact > 30 days before death had fewer ED contacts, secondary care hospitalizations, and readmissions than those with first SPC contact ≤ 30 days before death. When stratified by age, sex, and municipality type, these associations remained consistent across nearly all subgroups. In contrast, first SPC contact ≤ 30 days before death was associated with higher numbers of ED contacts and secondary care hospitalizations relative to both first SPC contact > 30 days before death and no SPC contact.

These findings align with earlier retrospective studies demonstrating that earlier integration of palliative care can reduce aggressive medical interventions near the EOL [8, 19, 23]. Seow et al. further showed that among patients with cancer, palliative care initiated earlier was associated with reduced acute hospital use, while Boddaert et al. reported that later or absent SPC was strongly associated with inappropriate EOL care, such as receiving chemotherapy during the last month of life or high use of ED or ICU services [13, 22]. The landmark randomized trial by Temel et al. similarly demonstrated that palliative care integrated earlier led to less aggressive EOL care compared to oncologic care alone [7]. Our results reinforce prior evidence suggesting that SPC initiated more than 30 days before death may mitigate high-intensity service use as patients approach their final weeks of life. Beyond the health

care service utilization, earlier SPC has shown to improve patient-centered outcomes. Randomized and observational studies that early palliative care improves QoL, mood and emotional well-being [7–10, 34]. For example, Bakitas et al. reported that early palliative care interventions experienced better mood and QoL compared to the usual care [34]. More recently, Greer at al. confirmed that early SPC contributes to both QoL and psychological adjustment [10]. To our knowledge, this is the first nationwide study to assess SPC timing and EOL service use among individuals with cancer as the confirmed underlying cause of death, addressing the key limitation of prior research, in which it was not known whether the patients actually died from cancer.

A critical finding from our study is that patients with first SPC contact ≤ 30 days before death had a higher prevalence of ED contacts and hospitalizations than those with first SPC contact > 30 days before death and those with no SPC contact. It is plausible that late referrals may be triggered by acute clinical deterioration or by symptom crises that necessitate hospitalization, a pattern documented in other settings [35–37]. Although patients with first SPC contact ≤ 30 days before death received outpatient and inpatient SPC services at rates similar to or even higher than those of patients with first SPC contact > 30 days before death, this intervention occurred too late to prevent acute service utilization. 

In this study, categorization of SPC timing was based on commonly used EOL quality indicators and prior literature [2, 3, 22], in which a 30-day threshold has been used to distinguish different timings of SPC initiation. This timeframe is also clinically relevant, as healthcare utilization is typically most intensive during the last month of life. Importantly, patients with first SPC contact > 30 days before death had a median duration of 122 days from first SPC contact to death, indicating that SPC was often initiated several months before death. 

Despite the high acute care use among patients with first SPC contact ≤ 30 days before death, SPC at any stage appeared to influence place of death. In our cohort, 52% of patients with first SPC contact ≤ 30 days before death and 60% of those with first SPC contact > 30 days before death died in hospital, compared with 83% of patients with no SPC contact. This suggests that even SPC initiated during the last month of life may help facilitate transfer from hospital to other care settings. The structured support provided through outpatient consultation services and inpatient SPC services, which were highly utilized among patients with first SPC contact ≤ 30 days before death, may have contributed to arranging discharges and supporting patients outside the secondary care setting in their final days. 

Our data also highlight the ongoing challenge of delayed SPC referral. Among all patients in the cohort, only 20% had first SPC contact > 30 days before death, meaning that SPC was initiated more than 30 days before death in only a minority of patients. This reflects a broader international issue, as SPC is often initiated in the last weeks of life, limiting the opportunity for patients to receive its full benefits for quality of life and emotional well-being [7, 12]. The median duration of SPC was 122 days among patients with first SPC contact > 30 days before death, compared with only 12 days among those with first SPC contact ≤ 30 days before death. This short duration among patients with first SPC contact ≤ 30 days before death suggests a missed opportunity for comprehensive, proactive care addressing not only physical symptoms but also psychological and spiritual needs, which are central components of palliative care. A systematic review by Johnson et al. demonstrated that the benefits of SPC increase with the duration of follow-up, with the most significant effects on QoL emerging after at least three months [12]. This underscores that referring patients to what is essentially EOL care during the final month of life means that those patients may miss months of potential benefit. 

The strengths of this study include its comprehensive nationwide coverage, encompassing all adults who died of cancer in Finland during 2019, which provides robust external validity within a publicly funded universal healthcare system. By linking multiple high-quality national registries, we were able to capture detailed information on healthcare utilization and SPC contacts across all levels of care among individuals who died from cancer. Nevertheless, certain limitations must be acknowledged. As a retrospective, registry-based study, causality between SPC timing and healthcare outcomes cannot be established. In addition, due to the retrospective design, some patients may have had late-stage diagnoses or rapid clinical deterioration, limiting the opportunity for earlier SPC referral. Although we adjusted for age, gender, and municipality type, residual confounding from unmeasured factors such as disease stage, performance status, socioeconomic background, and comorbidities may remain. Information on individual-level socioeconomic position (e.g., education or income) was not available in the registers used in this study. Recent evidence indicates that socioeconomic disparities can influence access to palliative care and the likelihood of dying at home among patients with cancer [34], suggesting that unmeasured social determinants may partly account for variation in SPC timing and service use. Furthermore, patient-reported outcomes, caregiver perspectives, and information on individual care preferences were not available, limiting our ability to assess the impact of SPC on QoL. In addition, the data were derived from 2019, since then, palliative care services in Finland have developed and service availability has improved. However, it is still too early to determine how these changes have influenced SPC utilization and EOL care patterns, and further research using more recent data is needed. Finally, while our findings are directly applicable to the Finnish context, their generalizability to healthcare systems with different structures, funding mechanisms, or palliative care models may be limited.

## Conclusion

Among patients dying of cancer, first SPC contact > 30 days before death, occurring a median of four months prior to death, was associated with reduced acute hospital resource use, including fewer ED contacts, hospitalizations, and readmissions. These observations suggest that SPC initiated earlier in the disease trajectory supports care trajectories that differ from those observed among patients with first SPC contact ≤ 30 days before death or no SPC contact.

## Data Availability

As the data is part of the larger dataset owned by the Finnish Institute for Health and Welfare (THL), the data generated during the current study are not publicly available. However, the data is available from the principal author Tiina Saarto upon reasonable request and with permission of the THL.

## Abbreviations

ED: Emergency department
EOL: End of life
SPC: Specialist palliative care
QoL: Quality of life
